# Characterization of M Cells in Tear Duct-Associated Lymphoid Tissue of Mice: A Potential Role in Immunosurveillance on the Ocular Surface

**DOI:** 10.3389/fimmu.2021.779709

**Published:** 2021-11-22

**Authors:** Yuki Oya, Shunsuke Kimura, Yutaka Nakamura, Narumi Ishihara, Shunsuke Takano, Ryo Morita, Mayumi Endo, Koji Hase

**Affiliations:** ^1^ Division of Biochemistry, Faculty of Pharmacy and Graduate School of Pharmaceutical Science, Keio University, Tokyo, Japan; ^2^ Precursory Research for Embryonic Science and Technology (PRESTO), Japan Science and Technology Agency, Saitama, Japan; ^3^ International Research and Developmental Center for Mucosal Vaccines, The Institute of Medical Science, The University of Tokyo, Tokyo, Japan

**Keywords:** tear duct-associated lymphoid tissue, microfold cell, RANKL, osteoprotegerin, IgA

## Abstract

The ocular mucosal tissues are exposed to potentially harmful foreign antigens in the air and tear fluid. The tear duct-associated lymphoid tissue (TALT) may contribute to immune surveillance in the eye region. Follicle-associated epithelium (FAE) of TALTs is classified as stratified squamous epithelium and consists of squamous epithelial cells arranged in layers on the basement membrane. In contrast, most mucosa-associated lymphoid tissue is covered by a monolayer of epithelium containing microfold (M) cells. Therefore, antigen uptake and the presence of M cells in TALT are not fully understood. The present study found that a small population of FAE cells in the TALT expressed intestinal M-cell markers, namely Sox8, Tnfaip2, GP2, and OPG. This cell population was identified as functional M cells because of their uptake capacity of luminal nanoparticles. In addition, RANKL, which is essential for M-cell differentiation, was expressed by stroma-like cells at the subepithelial region and its receptor RANK by the FAE in the TALT. The administration of RANKL markedly increased the number of Sox8^+^ M cells. In contrast, deficiency in OPG, an endogenous inhibitor of RANKL, increased the number of M cells in the TALT. These data demonstrate that the RANKL-RANK axis is essential for M-cell differentiation in the TALT. Furthermore, immunization *via* eye drops elicited the production of antigen-specific antibodies in tears, which was enhanced by RANKL administration. Thus, TALT M cells play an important role in the immunosurveillance of the eye region.

## Introduction

The ocular mucosal surface is exposed to potentially harmful foreign antigens, including pathogenic virus and pollen antigens in the air (1). The conjunctiva lines inside the eyelid and the surface of the sclera and is associated with the nasolacrimal duct, which carries tears to the nasal cavity *via* the lacrimal sac (2). Tears secreted by the lacrimal glands cover the ocular surface to drain the foreign antigens. Notably, tears contain a substantial amount of secretory IgA (S-IgA) that prevents bacterial and viral adhesion on the surface of the ocular mucosa and inactivates bacterial toxins.

S-IgA is mainly induced in mucosa-associated lymphoid tissue (MALT) composed of one or more lymphoid follicles found in various submucosal membrane sites of the body, such as the gastrointestinal tract nasopharynx, lung, and ocular mucosa (3). MALTs are responsible for inducing immune responses against mucosal antigens. A proportion of lymphoid tissue of the ocular mucosa is situated in the vicinity of the lacrimal sac, which can be found in humans and rodents and is referred to as tear duct associated lymphoid tissue (TALT) (4–7). TALT shares anatomical features with other MALTs and may play pivotal roles in immune surveillance and S-IgA induction against antigens in the tear fluid.

In the intestinal tract, Peyer’s patch is the inductive site for IgA responses. The follicle-associated epithelial cells (FAE) covering Peyer’s patches are characterized by the presence of microfold (M) cells, which are specialized intestinal epithelial cells that sample luminal antigens for mucosal immune surveillance (8–10). M cells actively transport macromolecules and microorganisms from the intestinal lumen into the subepithelial region *via* a transepithelial pathway known as antigen transcytosis. The transcytosed luminal antigens are subsequently phagocytosed by immature dendritic cells (DCs) in the subepithelial region. This causes the immature DCs to undergo maturation and, in turn, activate antigen-specific naive T cells. Thus, M-cell-dependent antigen transcytosis may have an important role in the induction of the mucosal immune response to specific antigens. Indeed, the absence of antigen uptake receptor glycoprotein 2 (GP2) in M cells attenuates the antigen-specific IgA antibody in feces of mice orally infected with *Salmonella enterica* serovar Typhimurium because of a decrease in bacterial uptake into Peyer’s patches (11).

Differentiation of M cells is triggered by receptor-activator of NF-κB ligand (RANKL), a TNF-family cytokine expressed by a stromal cell subset termed M-cell inducers in Peyer’s patches (12, 13). RANKL stimulation upregulates Spi-B and Sox8 responsible for the differentiation of functionally mature GP2^+^ M cells in the FAE of Peyer’s patches (14–17). These transcription factors are also essential for the differentiation of GP2^+^ M cells in nasopharynx-associated lymphoid tissue (NALT) (18). Therefore, mice lacking either Spi-B or Sox8 lacks mature M cells, leading to attenuated antigen uptake into Peyer’s patches. This results in reduced germinal center reactions and antigen-specific IgA responses. Notably, M cells abundantly produce Osteoprotegerin (OPG), a decoy receptor for RANKL, and hampers the RANKL-RANK interaction. Thus, OPG suppresses excessive M-cell differentiation in the intestine (19, 20). This is considered self-regulation machinery to control the number of M cells. Ablation of OPG increased M-cell numbers and eventually activated the commensal bacteria-specific IgA and IgG responses in the gut. These observations demonstrate that M cells contribute to the onset of the mucosal immune response.

Early works by electronmicroscopy and glycohistochemical staining with *Ulex europaeus* agglutinin I (UEA-I) have suggested the presence of M-like cells in the TALT (4). However, little is known about their functional properties and the molecular basis of M-cell differentiation in the TALT. Furthermore, unlike Peyer’s patches covered by a monolayer of epithelium, TALT of rodents is overlaid by stratified squamous epithelium that forms a robust physical barrier. It remains to be determined whether there are *bona fide* M cells, whose functional property and molecular markers are equivalent to those of the intestinal M cells, in stratified squamous epithelium.

In the present study, we detected Sox8-expressing M cells with the simultaneous expression of Spi-B, Tnfaip2, GP2, and OPG in the FAE of TALT. These cells have a high uptake capacity for luminal nanoparticles. The administration of RANKL significantly increased the number of M cells in the TALT FAE and increased the antigen-specific IgA antibody in tears. These results indicate that antigen uptake by M cells in TALT causes an immune response in the eye region.

## Material and Methods

### Animals

Six- to fifteen-week-old male BALB/cCrSlc (BALB/c) mice and C57BL/6J mice were purchased from Japan SLC and maintained under specific pathogen-free conditions. *Opg* ^−/−^ mice on a C57BL/6J background were described previously (21), and C57BL/6J WT mice were used as a control of *Opg* ^−/−^ mice. All experiments involving the use of animals were performed under protocols following the Guidelines for Animal Experimentation at the Animal Use Committee at the Keio University approved all animal experiments.

### Isolation of Nasolacrimal Duct

The preparation of the nasolacrimal duct was illustrated in **Supplementary Figure 1**. Mice were euthanized by cervical dislocation, and the heads were separated. After removing the epidermis and lower jaw, the head was amputated in half along the midline by scissors. The incisor and hard palate were removed. Frontal, premaxillary, maxillary, and lacrimal bones were exposed by a precision tweezer. Subsequently, the maxillary bone was carefully amputated with scissors not to damage the lacrimal bone and the associated lacrimal sac. The nasolacrimal duct was then exposed by carefully peeling off the face from the bones. The nasolacrimal ducts with lacrimal sac in which TALT is present were isolated from both eyes.

### Whole-Mount Immunohistochemical Staining

The nanosacrimal duct was dissected around tissue and fixed with 4% paraformaldehyde (PFA; pH 7.4) for 24 h. Fixed tissues were dehydrated with methanol. To block endogenous peroxidase activity, tissues were incubated with 0.3% hydrogen peroxide for 30 min. Tissues were further incubated in 0.1% Triton X-100 (Nacalai), and 0.1% skim milk (Nacalai) in phosphate-buffered saline (PBS; pH 7.2) followed by the incubation with anti-B220 antibodies at 4°C overnight. To detect the primary antibodies, tissues were incubated with peroxidase (HRP)-conjugated secondary antibodies at 4°C overnight. The HRP signal was visualized with Peroxidase Stain DAB Kit (Nacalai). The specimen was observed and was captured as images by stereomicroscope M125 (Leica).

### Immunofluorescent Staining

In most experiments, isolated nasolacrimal ducts and lacrimal sacs were fixed with 4% PFA for 24 h. When decalcification was required for staining, the mice head was immersed in the 4% PFA for 24 h and then decalcified with 5% EDTA for 2-3 weeks at 4°C. The fixed tissues were placed in a 30% sucrose solution overnight at 4°C, embedded in OCT compound (Sakura Finetek), and quickly frozen in liquid nitrogen. Frozen sections were prepared as approximately 15-20-μm thick, were mounted on MAS-coated slide glass, and air-dried (Matsunami glass). To detect Spi-B, the tissue sections were subjected to antigen retrieval by heating in a microwave oven in Tris-HCl buffer with EDTA solutions (pH 9.0). For triple immunofluorescence staining with antibodies of RANKL, RANK, and Tnfaip2, the isolated tissues were immediately embedded in the OCT compound without any fixation. Subsequently, the fresh frozen sections were prepared, mounted on MAS-coated slide glass, and fixed with 4% PFA for 15 min.

After pretreatment with 0.3% Triton X-100 in PBS for 30 min and preincubation with 10% normal donkey serum, the sections were incubated with the primary antibodies at room temperature overnight. The sites of the antigen-antibody reaction were detected by incubation for 2 h at room temperature, with secondary antibodies corresponding to the animal species of the primary antibodies. For nuclear staining, Hoechst 33342 (Life Technologies) was used with the secondary antibodies. The specimens were observed using a confocal laser microscope FV3000 (Olympus) after mounting with ProLong™ Gold Antifade Mountant (Thermo Fisher Scientific).

The antibodies for immunofluorescent staining are listed in **Supplementary Table 1**.

### Eye Drop Instillation of Fluorescence Nanoparticles

Before experimental manipulation, mice were anesthetized by i.p. injection of a combination anesthetic prepared with 0.3 mg/kg of medetomidine, 4.0 mg/kg of midazolam, and 5.0 mg/kg of butorphanol (22). Fluorescent nanoparticles (200 nm in diameter; Polysciences, Inc.) were dropped into the ocular surface of BALB/c mice. At 30 min after administration of atipamezole, mice were euthanized, and isolated lacrimal sacs were subjected to immunohistochemical experiments.

### Fluorescence *In Situ* Hybridization

FISH was performed using the Quantigene View RNA ISH Cell Assay (Affymetrix) with slight modifications in the fixation (18). Briefly, fresh specimens were immediately embedded in the OCT compound. Frozen sections were mounted on MAS-coated slide glass and fixed with 4% PFA for 15min. The sections were pretreated with a detergent solution for 10min at room temperature. Subsequent processes were performed following the manufacturer’s protocol. Specific oligonucleotide probe sets against *Tnfrsf11* (Cat: VB1-13707) were purchased from Affymetrix, Inc.

### RANKL Administration

The primers 5′-CACCCCCGGGCAGCGCTTCTCAGGA GCT-3′ and 5′-GAGACTCGAGTCAGTCTATGTCCTGAAC-3′ (Sigma Genosys) were used for polymerase chain reaction (PCR) to amplify a cDNA clone of mouse RANKL. The PCR fragment was subcloned into the pGEX-4T-2 vector (GE Healthcare) after digestion by SmaI and XhoI. The construct was transformed into the BL21 *Escherichia coli* strain for glutathione-S-transferase (GST) fusion protein expression. The culture was induced with 0.1mM isopropyl β-D-1-thiogalactopyranoside for 16 h at 20°C, and GST-RANKL was purified from the bacterial lysate by affinity chromatography on a Glutathione-Sepharose 4B (GE Healthcare) followed by dialysis against multiple changes of PBS. Recombinant GST used as a control was prepared by the same method using an empty pGEX-4T-2 vector. Purified protein was administered to mice by intraperitoneal injections at a dose of 10 mg/kg/day for 3 days. After 24h from the last administration, the mice were sacrificed and subjected to immunostaining for cryosection.

To determine the purities of the prepared GST and GST-RANKL, an aliquot (1.25µg) of each protein was separated by SDS-PAGE and stained with CBB Stain One (Nacalai) at room temperature for 15 min. The purities of both proteins were determined by densitometry of CBB staining (**Supplementary Figure 2**). The purities of both proteins were more than 90%. In addition, the endotoxin levels in GST and GST-RANKL protein solutions, which were measured by ToxinSensor Chromogenic LAL Endotoxin Assay Kit (GenScript), were 350 and 666 EU/mL, respectively.

### Quantitative Reverse Transcriptase PCR

The nasolacrimal duct was exposed as described above, and the regions including TALTs were prepared from both eyes. Total RNA of TALTs isolated from mice were extracted using TRIzol (Life Technologies). First-strand cDNA synthesis was completed using ReverTra Ace qPCR RT Master Mix with gDNA Remover (TOYOBO). Quantitative PCR reactions were conducted in CFX-connect (Bio-Rad) using a SsoAdvanced Universal SYBR Green Supermix (Bio-RadHercules). The specific primers used in these assays are shown in **Supplementary Table 2**.

### Administration of Anti-RANKL Antibody

Rat anti‐mouse RANKL‐neutralizing monoclonal antibody (5 mg/kg, clone: OYC1, Oriental Yeast Co.) were intraperitoneally injected into BALB/c mice twice every four days. After four days from the second administration, mice were euthanized, and the heads were removed and subjected to immunofluorescent staining.

### Immunization

Under anesthesia, 100 µg ovalbumin (OVA; Sigma-Aldrich) and 2 µg cholera toxin (CT; FUJIFILM Wako Pure Chemical Industries) suspended in 5 µL PBS per eye were dropped weekly for three consecutive weeks by micropipette (23). After seven days from the last administration, the mice were euthanized and subjected to lectin staining, flow cytometry, and ELISA. In a separate experiment, BALB/c mice were intraperitoneally injected with GST or GST-RANKL for three consecutive days before the ocular immunization.

### ELISA for Detection of OVA-Specific Antibodies

The immunized mice were anesthetized, and blood samples were collected through cardiac puncture. Serum samples were isolated from blood by centrifugation. Tear-wash samples were obtained by lavaging with 10 µL PBS per eye. ELISA plates (Thermo Fisher Scientific) were coated with OVA (1 mg/ml) in PBS and incubated overnight at 4 °C. Blocking was performed with 2% BSA (Nacalai) in PBS, and 2-fold serially diluted samples were applied to plates. HRP-conjugated goat anti-mouse IgG or IgA antibodies (Bethyl Laboratories) were added to each well and incubated for 1 h at room temperature. For color development, 1-Step™ Ultra TMB-ELISA Substrate Solution (Thermo Fisher Scientific) was used. Then, plates were measured at 450 nm on the Infinite 200 PRO multimode plate reader (Tecan Group Ltd.) after adding 1.2M H_2_SO_4_ to stop the reaction.

### Flow Cytometry

Single leukocyte suspensions of cervical lymph nodes (cLNs), submandibular lymph nodes (smLNs), and the nasolacrimal duct including TALTs from both eyes were prepared by mechanically disrupting tissues through 100-μm nylon mesh cell strainers (Greiner Bio-One) in 2% FCS RPMI1640 media (MP Biomedicals; nacalai) including HEPES (nacalai) and 1% Penicillin-Streptomycin Mixed Solution (nacalai). Isolated cells were incubated with TruStain FcX™ PLUS (anti-mouse CD16/32 antibody; 1:100; BioLegend; 156604) to block non-specific reactions and subsequently stained by using specific antibodies. After surface staining, dead cells were stained by 7-AAD Viability Staining Solution (1:200; Biolegend; 420404). Flow cytometric analyses were performed using an LSRII (BD Biosciences). The antibodies used for flow cytometry are shown in **Supplementary Table 3**.

### Quantitative Image Analysis

For quantification of sections, at least three cryosections of TALT FAE from each mouse were subjected to immunofluorescence analysis with a BZ-X810 All-in-One fluorescence microscope (Keyence). The area of TALT FAE and the number of Sox8^+^ cells were measured by BZ-X800 Analyzer software (Keyence).

### Statistical Analysis

Differences between mean values for two or more groups were analyzed by Student’s *t*-test, Mann-Whitney U test, one-way ANOVA with Tukey- Kramer test, two-way ANOVA, or Kruskal-Wallis test, respectively, using Prism (GraphPad Software).

## Results

### The Isolation of TALT From the Nasolacrimal Duct

**Figure 1:**
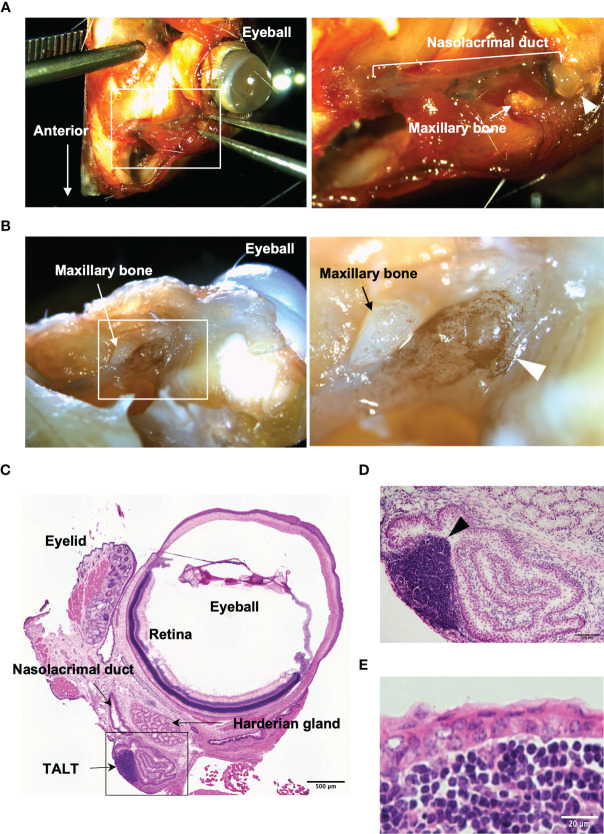
Macroscopic isolation of TALT from mice head. **(A)** A stereomicroscopic image of the face around the eye of the mouse is shown. The nasolacrimal duct contains Evans blue that is a blue staining solution given *via* eye drops. The right panel shows the highly magnified image of the area surrounded by the white line in the left panel. **(B)** A whole-mount immunohistochemical image of the same sample in panel **(A)** is shown. B lymphocytes were detected with HRP labeled anti-B220 antibody (brown). The right image is an enlarged view of the squared area in the left panel. Each arrowhead in panels **(A, B)** shows a lacrimal sac of the nasolacrimal duct. **(C–E)** Hematoxylin and eosin-stained sections of excised ocular mucosal tissue. Panel **(D)** shows a highly magnified image of the lacrimal sac area surrounded by a black line in panel **(C)** An arrowhead indicates a lymphoid follicle of TALT. TALT is covered with stratified squamous epithelium. n = at least 5 animals. Bars: 500 µm **(C)**, 100 µm **(D)**, 20 µm **(E)**.

TALT is attached to the nasolacrimal sac, which is located in a bone-enclosed area (7). We initially confirmed the macroscopic position of TALT and developed a method for isolating fresh TALT from the head bone of mice (**Figures 1A, B** and **Supplementary Figure 1**). Macroscopic observation showed a ductal structure with a dilated end on the eye side and the other end entering the bone (**Supplementary Figure 1**). Since this ductal structure contained Evans blue dye instilled from the eye in the lumen, we concluded that this ductal structure was the nasolacrimal duct that connects from the lacrimal canaliculi to the nasal cavity (**Figure 1A**). The dilated region was attached with a thin bone, the lacrimal bone, and was located underneath the maxillary bone, indicating a lacrimal sac (**Supplementary Figure 1**). Whole-mount immunostaining with anti-B220 antibody revealed brown-stained B-cell aggregates associated with the lacrimal sac (**Figure 1B**). Hematoxylin and eosin staining of thin sections of the region containing the nasolacrimal duct and an eye showed a lymphocyte aggregation of TALT in the lacrimal sac near the eyeball and Harderian gland (**Figures 1C, D**). The follicle of TALT was covered with the stratified squamous epithelium, which was composed of two- or three-layer cells (**Figure 1E**).

### GP2^+^ Sox8^+^ Tnfaip2^+^ Cells in TALT Are Functional M Cells

**Figure 2:**
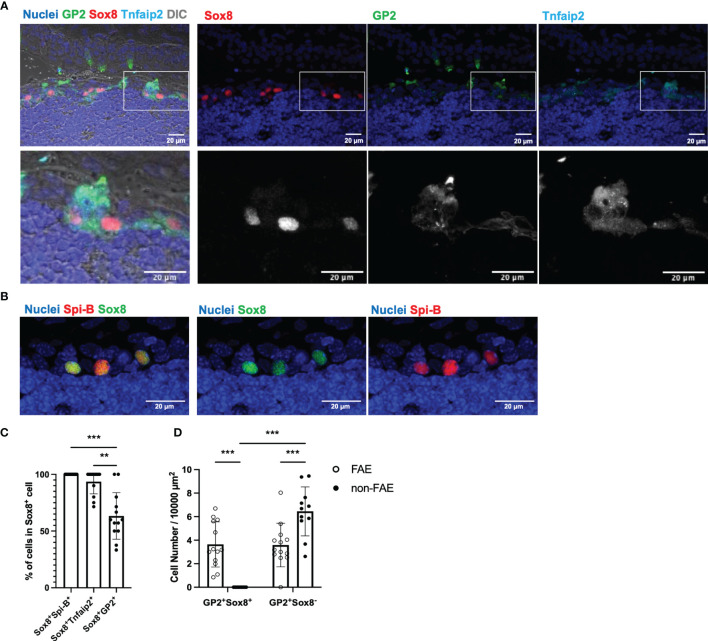
Confocal microscope images of TALT FAE. **(A)** Multicolor immunofluorescence images of GP2 (green), Sox8 (red), and Tnfaip2 (cyan) were shown. Lower panels are higher magnification images of the area illustrated at the upper panel. Each channel is shown separately in each color with nuclei (upper panel) and in grayscale (lower panel). DIC, differential interference contrast. **(B)** Fixed cryosections, including TALT, were heated in Tris-HCl buffer with EDTA solutions (pH 9.0) before immunostaining of Spi-B for retrieving antigens. Spi-B (red) Sox8 (green) double-positive cells are found in TALT FAE. Nuclei were stained with Hoechst 33342 (blue). Bars: 20 µm. Representative images of those obtained from at least 3 animals are shown. Images were acquired by confocal microscopy. **(C)** Quantification of Sox8^+^ Spi-B^+^, Sox8^+^ Tnfaip2^+^ and Sox8^+^ GP2^+^ cells in Sox8^+^ cells of TALT FAE. Individual symbols represent the frequency of each cell population in a cryosection. Bars represent the mean ± standard deviation. ***P < 0.001, **P < 0.01 (Kruskal-Wallis test, n = 5 animals). **(D)** The number of GP2^+^ Sox8^+^ or GP2^+^ Sox8^-^ cells in FAE or non-FAE were measured. Bars represent the mean ± standard deviation. ***P < 0.001 (two-way ANOVA test, n = 5 animals).

We subsequently asked whether M cells are localized in the stratified squamous epithelium of TALT. Immunofluorescence analysis of the TALT revealed Sox8^+^ cells that simultaneously expressed GP2, Tnfaip2, and Spi-B (**Figures 2A, B**). Quantification data manifested that almost all Sox8^+^ cells were positive for Spi-B and Tnfaip2 (**Figure 2C**). On the other hand, the frequency of GP2-coexpressing cells in Sox8^+^ cells was 63.32 ± 5.70% (means ± S.E.M.) because only mature, but not immature, M cells upregulate GP2 (24).

GP2 is a reliable M-cell marker in the intestine; however, in the mouse head, secretory cells, including goblet cells and acinar cells in mucous glands, highly express GP2, as we reported previously (25, 26). In fact, GP2^+^ Sox8^+^ cells were found only in FAE of TALT, whereas GP2^+^ Sox8^-^ cells were both in FAE and outside of FAE (**Figure 2D**). Similarly, UEA-I^+^ Sox8^-^ cells were found outside of FAE (**Supplementary Figure 3**). Thus, it is unlikely that GP2^+^ Sox8^-^ cells and UEA-I^+^ Sox8^-^ cells are M cells in the nasolacrimal duct and the lacrimal sac.

We further confirmed that Sox8^+^ cells expressed other M-cell-associated molecules, including Marksl1 (27), CCL9 (28), CCL20 (29, 30), Uromodulin (15, 16), and Aif1 (31), in addition to the epithelial marker, EpCAM (**Supplementary Figure 4**). This indicates that Sox8^+^ cells in TALT are most likely equivalent to M cells of Peyer’s patch, NALT, and inducible bronchus-associated lymphoid tissue (iBALT) (32).

**Figure 3:**
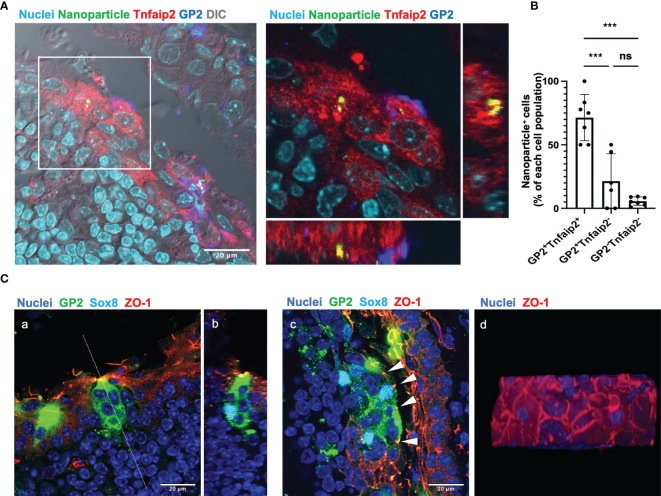
GP2^+^ Tnfaip2^+^ cells take up the fluorescent nanoparticles instilled by eye drop. **(A)** GP2 (blue) Tnfaip2 (red) -positive cells take up 200 nm nanoparticles (green) dropped into the eyes of BALB/c mice under anesthesia as described in the method. The right panel shows a higher magnification of the area outlined in the left panel. Representative images of those obtained from at least 3 animals are shown. **(B)** Quantification data of the uptake ability of GP2^+^ Tnfaip2^+^ cells, GP2^+^ Tnfaip2^-^ cells, and GP2^-^ Tnfaip2^-^ cells. Data represent the percentage of bead^+^ cells in each cell population. Bars represent the mean ± standard deviation. ***P < 0.001, ns not significant (Kruskal-Wallis test, n = 4 animals). **(C)** Immunofluorescence analysis of thick cryosections (40 µm) shows the expression of ZO-1 (red) in the outer layer of the TALT FAE. Green is GP2 and cyan is Sox8. Panel b shows a sliced image along the dotted line in panel a, reconstructed from consecutive confocal microscope images. Arrowheads in panel c show the tight junction in FAE of TALT. Panel d shows a top view of the FAE of the reconstructed 3D image. Representative images of those obtained from at least 4 animals are shown. Nuclei were stained with Hoechst 33342 (blue). Bars: 20 μm.

We evaluated the uptake ability, which is the primary function of M cells, by eye drop instillation of fluorescent nanoparticles with a diameter of 200 nm. Confocal microscopy demonstrated that the nanoparticles were incorporated by GP2^+^ Tnfaip2^+^ cells of the TALT FAE (**Figure 3A**). Quantitative image analysis showed that GP2^+^ Tnfaip2^+^ cells have a higher uptake ability than GP2^+^ Tnfaip2^-^ cells and GP2^-^ Tnfaip2^-^ cells (**Figure 3B**). These data illustrate that Sox8^+^ GP2^+^ Tnfaip2^+^ cells in TALT are *bona fide* M cells with antigen uptake capacity.

Because the FAE of TALT is composed of stratified squamous epithelium, we analyzed which layer of the epithelial cells form tight junctions. Immunostaining with ZO-1 manifested that tight junctions are formed in the most outer layer of the stratified epithelium covering TALT (**Figure 3C**). M cells marked with GP2 and Sox8 traverse the stratified epithelial layers from the mucosal surface to the basement membrane. Interestingly, GP2-positive M cells in TALT appeared to possess several cell nuclei; however, a single nucleus is positive for Sox8. Given that Sox8 is a master transcription factor of M cells, the other Sox8-negative nuclei may be intraepithelial lymphocytes associated with the M-cell pockets (8).

### RANKL-RANK Axis Induces M-Cell Differentiation in TALT

**Figure 4:**
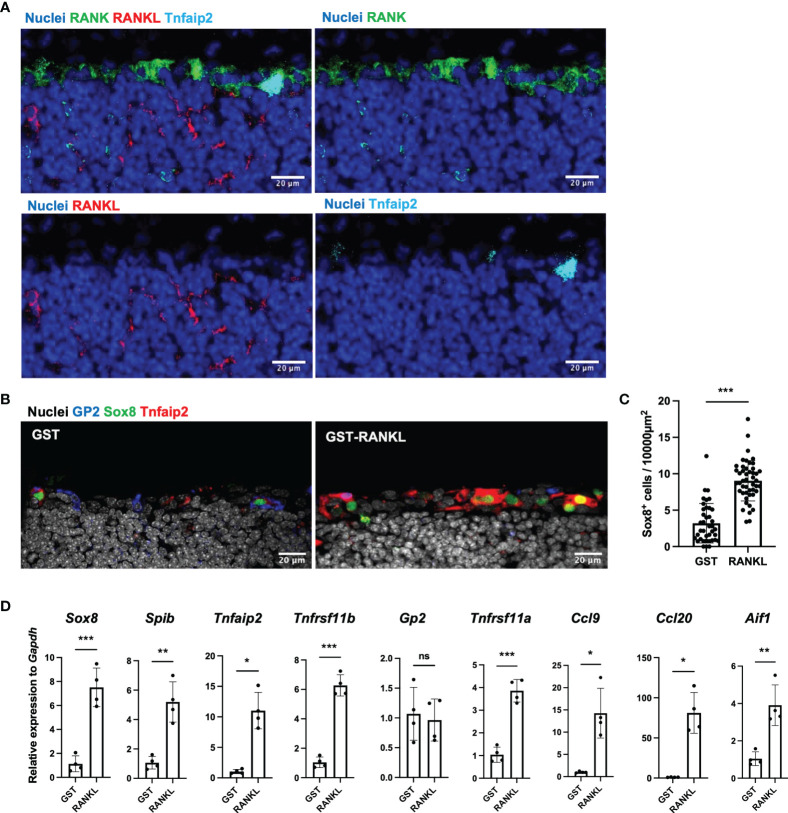
RANKL is an inducer of M cells in the TALT. **(A)** Cryosection of the TALT was immunostained for RANK (green), RANKL (red), and Tnfaip2 (cyan). Nuclei were stained with Hoechst 33342 (blue). Bars: 20 µm. Representative images of those obtained from at least 4 animals are shown. **(B)** Confocal microscopic images show the expression of GP2 (blue), Sox8 (green), and Tnfaip2 (red) in the TALT FAE in the BALB/c mice administered 10 mg/kg GST as control or GST–RANKL daily for 3 days. Nuclei were stained with Hoechst 33342 (gray). Bars: 20 µm. **(C)** Quantification of the frequency of Sox8^+^ cells in the FAE of TALT. Data were obtained from a single experiment. ***P < 0.001 (Student’s t-test, n = 3 animals, respectively). The individual dot represents the number of Sox8^+^ cells per 10,000 µm^2^ of FAE. **(D)** Quantitative PCR analysis of M-cell associated genes in TALT of BALB/c mice injected with GST-RANKL or GST as a control. Data were normalized to *Gapdh* expression and are presented relative values to the expression in the TALT of GST-treated mice. Data were obtained from 4 mice in each group from a single experiment. Bars represent the mean ± standard deviation. ***P < 0.001, **P < 0.01, *P < 0.05, ns, not significant (*Sox8, Spib, Tnfrsf11b, Gp2, Tnfrsf11a* and *Aif1*: Student’s t-test, *Tnfaip2, Ccl9 and Ccl20*: Mann-Whitney U test. n = 4 animals, respectively).

To investigate the molecular basis of M-cell differentiation in TALT, we performed immunofluorescent staining for RANKL and its receptor, RANK. Consistent with Peyer’s patches, RANKL was expressed by stromal cells in the subepithelial region of TALT, whereas RANK was located throughout the FAE (**Figure 4A**). Similarly, fluorescence *in situ* hybridization analysis detected RANKL mRNA (*Tnfsf11*)-positive cells in the subepithelial region (**Supplementary Figure 5**).

To further confirm the importance of the RANKL-RANK signal in M-cell differentiation, we administrated GST-RANKL intraperitoneally for three days to wild-type mice. The GST-RANKL administration increased the number of GP2^+^ Sox8^+^ Tnfaip2^+^ cells in TALT (**Figure 4B**). Quantitative image analyses indicated that the number of Sox8^+^ cells increased approximately 2.8-fold by GST-RANKL administration compared with the GST control (**Figure 4C**). Quantitative PCR analysis confirmed the RANKL-dependent upregulation of most of the M-cell-marker genes (**Figure 4D**). In contrast, *Gp2* expression was comparable to the control, which may result from the high expression of GP2 in the goblet cells of the head mucosa (25). We further treated mice with an intraperitoneal injection of anti-RANKL neutralizing antibody. This treatment almost wholly eliminated the M cells in the Peyer’s patches and tended to reduce the number of TALT M cells (**Supplementary Figure 6**).

**Figure 5:**
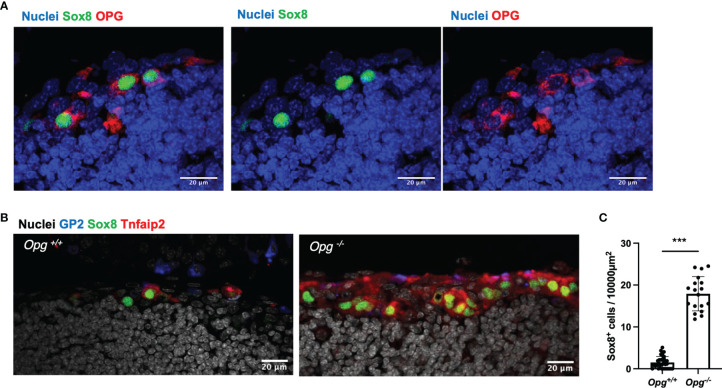
OPG suppresses the differentiation of M cells in TALT. **(A)** Sox8 (green) positive cells express OPG (red) in TALT FAE of BALB/c mice. Nuclei were stained with Hoechst 33342 (blue). Bars: 20 µm. Representative images of those obtained from at least 3 animals are shown. **(B)** Immunofluorescence images of GP2 (blue), Sox8 (green), and Tnfaip2 (red) in the FAE of TALT in the *Opg^+/+^* or *Opg ^-/-^* mice. Nuclei were stained with Hoechst 33342 (gray). Bars: 20 µm **(C)** Quantification of the frequency of Sox8^+^ cells in the FAE of TALT. Data were presented as the mean ± standard deviation. ***P < 0.001 (Mann-Whitney U test, n = 3 animals, respectively). The individual dot represents the number of Sox8^+^ cells per 10,000 µm^2^ of FAE.

Immunostaining of TALT also showed expression of OPG in Sox8^+^ cells (**Figure 5A**), suggesting that OPG-dependent self-regulation of M-cell differentiation may also be the case in TALT. To test this possibility, we analyzed TALT FAE from OPG-deficient mice. As expected, the number of M cells in these mice was remarkably increased compared with that of the control mice (**Figures 5B, C**). Collectively, these results illustrate that the RANKL-RANK signaling is essential for M-cell differentiation in TALT, whereas M-cell-derived OPG limits the number of M cells.

### TALT M Cells Contribute to Antigen-Specific Immune Response

**Figure 6:**
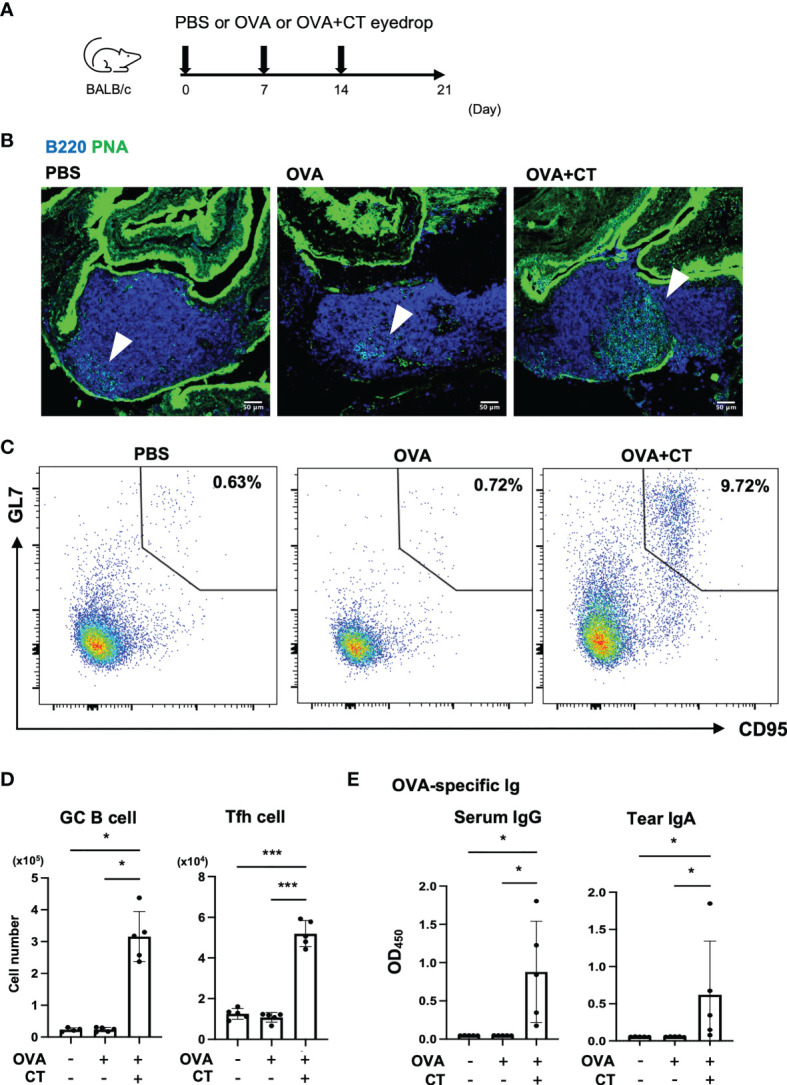
Eye drop instillation of ovalbumin with cholera toxin adjuvant elicits a germinal center reaction in TALT and draining lymph nodes. **(A)** Immunization scheme of eye-drop immunization of BALB/c mice with PBS, OVA, or OVA with CT. **(B)** Confocal microscopic analysis of TALT sections stained with a lectin, PNA (green), and anti-B220 antibody (blue). Arrowheads show germinal center (PNA^+^ B220^+^ cells). Bars: 50 µm. **(C)** Expression of CD95 and GL7 on TALT cells pooled from the 4 or 5 mice. Numbers in outlined areas show the percent of germinal center (GC) B cells (CD95^+^ GL7^+^ cells) in CD3e^-^ B220^+^ lymphocytes in TALT. **(D)** The number of GC B cells and Tfh cells (CXCR5^+^ PD-1^+^ in CD4^+^ CD8^-^ T cell) in the cervical and submandibular lymph nodes (cLN and smLN) was quantified by flow cytometry at 1 week after the final immunization. Gating strategies for the flow cytometry analyses were shown in **Supplementary Figure 7**. **(E)** OVA-specific serum IgG levels and tear IgA levels were measured by ELISA. Data were obtained from a single experiment. Bars represent the mean ± standard deviation. ***P < 0.001, *P < 0.05 (GC B cell, OVA-specific serum IgG and tear IgA: Kruskal-Wallis test, Tfh cell: one-way ANOVA by Tukey’s test, PBS: n = 4 or 5, OVA: n = 5, OVA + CT: n = 5 animals).

To clarify the contribution of TALT M cells to mucosal immunity of the eye, we immunized mice with OVA plus CT by instillation for three consecutive weeks (**Figure 6A**). Lectin-fluorescence staining with PNA and flow cytometry confirmed the expansion of germinal center (GC) B cells in TALT (**Figures 6B, C**). The expansions of GC B cells and T follicular helper (Tfh) cells were also evident in the regional lymph nodes of the head, namely, the smLN and cLN (**Figure 6D** and **Supplementary Figure 7**). Accordingly, the amount of OVA-specific serum IgG and tear IgA increased after the ocular immunization (**Figure 6E**).

**Figure 7:**
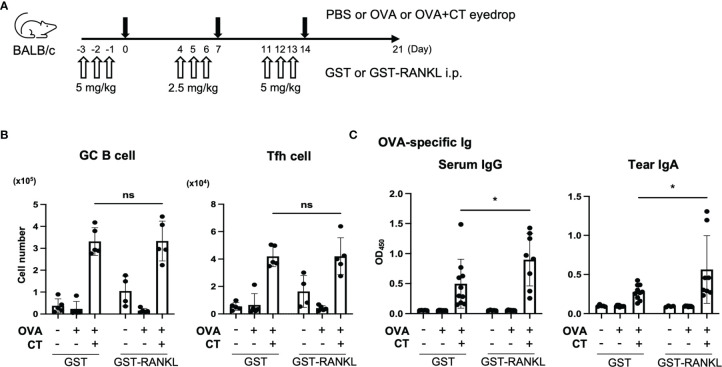
Administration RANKL enhances immune response in the eye region. **(A–C)** BALB/c mice were intraperitoneally injected GST or GST-RANKL and immunized by eye drops as illustrated in panel **(A)** (first and third week: 5 mg/kg, second week: 2.5 mg/kg). **(B)** The number of GC B cells and Tfh cells in the cLN and smLN was quantified by flow cytometry. Representative data are shown from two independent experiments. ns not significant (two-way ANOVA, PBS/GST: n = 5, OVA/GST: n = 5, OVA + CT/GST: n = 5, PBS/GST-RANKL: n = 4, OVA/GST-RANKL: n = 5, OVA + CT/GST-RANKL: n = 5 animals). **(C)** Quantification of OVA-specific serum IgG and tear IgA by ELISA. Data were pooled with two independent experiments and are presented as the mean ± standard deviation. *P < 0.05 (two-way ANOVA, PBS/GST: n = 5, OVA/GST: n = 10, OVA + CT/GST: n = 10, PBS/GST-RANKL: n = 4, OVA/GST-RANKL: n = 9, OVA + CT/GST-RANKL: n = 8 animals from two independent experiments).

In the subsequent experiment, the mice received systemic administration of GST-RANKL prior to the immunization to increase TALT M cells (**Figure 7A**). The GST-RANKL treatment significantly increased the amount of OVA-specific tear IgA as well as serum IgG, whereas the number of GC B cells and Tfh cells were unchanged in the smLN and cLN at three weeks post-immunization (**Figures 7B, C** and **Supplementary Figure 8**). These observations imply that M-cell-dependent antigen uptake in TALT may facilitate both local and systemic immune responses after ocular immunization.

## Discussion

TALT, surrounded by the maxillary and lacrimal bone, has been an unexplored region by thorough histological and immunological analysis because of the difficulty in macroscopic identification, isolation, and sample preparation. In the present study, we developed a new technique to isolate a nasolacrimal duct with TALT from mouse heads, enabling immunostaining of TALT without the time-consuming decalcification process.

Multicolor immunofluorescence imaging revealed that GP2^+^ Sox8^+^ Tnfaip2^+^ cells in the TALT FAE are the equivalent of intestinal M cells because they absorb luminal materials and express antigen-uptake-related molecules (i.e., GP2, Aif1, and Uromodulin) as well as transcription factors essential for regulating these molecules (i.e., Spi-B and Sox8). GP2 is expressed on the apical surface of M cells and interacts with FimH, a major component of the type 1 pilus on the outer membrane of a subset of gram-negative enterobacilli, such as *E. coli* and *Salmonella enterica* (11). GP2 acts as a receptor that interacts with these enteric bacteria, facilitating uptake from M cells to activate the antigen-specific immune response. Aif1, a cytosolic protein that facilitates F-actin polymerization, is associated with M-cell transcytosis by regulating the activation of β1 integrin, a potential receptor during *Yersinia enteroclitica* invasion (31). Similarly, Uromodulin functions as a receptor for the uptake of the L-92 strain of *Lactobacillus* (33). Meanwhile, Spi-B and Sox8 regulate the expression of these functional molecules required for antigen uptake by M-cells (14–17). Most of these molecules are commonly expressed in the M cells not only of TALT and Peyer’s patches but also of NALT and iBALT, suggesting that antigen uptake by M cells and their molecular mechanisms are fundamental elements shared among the MALTs (9, 18, 32).

We previously defined functionally immature M cells and mature M cells characterized by GP2 expression and high antigen-uptake capability in the gut-associated lymphoid tissue (24). However, the present study exhibited that Sox8^-^ GP2^+^ cells were scattered outside of TALT FAE, indicating that single staining of GP2 is insufficient to detect M cells in the ocular tissue. Based on these observations, we propose that double staining of GP2 together with an early M-cell marker: Sox8, Spi-B, or Tnfaip2, is essential to identify fully differentiated M cells. In conclusion, the combinatorial detection of several M-cell-associated molecules such as Sox8, Spi-B, GP2, and Tnfaip2 is a reliable strategy to identify M cells in the various mouse mucosal tissue. Considering that Sox8, Spi-B, and GP2 are expressed in human M cells of Peyer’s patch or RANKL-stimulated primary human enteroids (11, 14, 17, 34), this detection method could apply to human M cells.

The ocular surface is continuously exposed to microorganisms, including various pathogens from the external environment. Interestingly, certain types of ocular pathogens have been reported to be taken up by M cells or translocated into the body *via* M cells. For instance, *E.coli* is the most common pathogen causing neonatal conjunctivitis (35, 36). As mentioned above, M cells take up *E.coli* in a GP2-dependent manner (11). Furthermore, *Mycobacterium tuberculosis* causes ocular tuberculosis (37), and *Pseudomonas aeruginosa* is the most common Gram-negative organism causing bacterial keratitis (38). These pathogens have also been known to exploit M cells for invasion (39, 40). However, it remains unknown about the contribution of TALT M cells to the establishment of ocular infection by these microorganisms and the induction of mucosal immune response to the pathogens. Further investigations on these issues will provide new insight into infection and host defense machinery on the ocular surface.

In TALT, RANKL-positive cells resided underneath the FAE, and RANK was expressed in FAE cells. This positional relationship of RANKL and RANK is similar to that in Peyer’s patches, cecal patches, and NALT (12, 13, 18). In addition, the administration of RANKL increased the number of M cells in the TALT. The observed increase in M-cell number was also confirmed in the deficiency of OPG. These data suggest that RANKL is a common inducer of M-cell differentiation in the TALT, NALT, and gut-associated lymphoid tissue. Like Peyer’s patches and cecal patches, TALT M cells may regulate their numbers by producing OPG (19, 20). In contrast, however, anti-RANKL neutralizing antibody did not inhibit M-cell-differentiation completely in the TALT even under the condition that M cells were almost eliminated in Peyer’s patches. It is, therefore, possible to speculate that other factors besides RANKL may be involved in M-cell differentiation in TALT; however, further studies will be required to prove this speculation.

Unlike GALT and NALT, TALT in rodents is covered with the non-keratinized stratified squamous epithelium, which forms a robust physical barrier against foreign antigens. Thus far, it has been well documented that Langerhans cells extrude their dendrites out of the stratified squamous epithelium through the tight junctions to directly sample antigens on the skin surface (41). Conjunctival DCs may also possess a similar function (42). Our study manifested that M cells are also present in the stratified squamous epithelium and may constitutively transport mucosal antigens to the immune system across multilayered epithelial cells. However, TALT covered by non-keratinized epithelium is a morphological feature specific for rodents, and thus, the results of our study may not be directly extrapolated to human TALT studies. Nonetheless, the human palatine tonsils, lingual tonsils, vagina, and endometrium are covered with non-keratinized stratified squamous epithelium, suggesting that they may take up luminal macromolecules *via* M cells (43).

The M cell represents a possible immunization route for the mucosal vaccine because it is an effective portal site for environmental antigens and infectious materials (44). We confirmed that eye drop instillation of antigens efficiently activated the germinal center reaction in TALT and the regional lymph nodes. Furthermore, administration of RANKL increased the number of M cells in the TALT and enhanced the antigen-specific IgA and IgG production in the tear and serum, respectively. Of note, GC B cells in the regional lymph nodes remained unchanged in the RANKL-treated group at three weeks post-immunization. Considering that GC reaction should precede antibody production, the analyses at earlier time points (e.g., 1 and 2 weeks after immunization) may be necessary to determine the influence of RANKL on the GC reaction. Our data suggest that RANKL may be a potential vaccine adjuvant that effectively induces mucosal immune vaccines. However, we do not exclude the possibility that the administration of RANKL promotes the survival of conventional DCs and enhances the immune response through T-cell priming and activation (45). Nevertheless, antigen uptake by M cells is the first step in the mucosal immune response, and increased uptake may play a central role in initiating a series of mucosal immune responses.

In the eye region, there is another MALT termed conjunctiva-associated lymphoid tissue (CALT). Like TALT, CALT is also covered by the stratified squamous epithelium containing M-like cells (23). Whereas CALT is rarely found in the nictitating membrane of mice at the steady state, stimulation with microbes or OVA/CT induces CALT (46). CALT and TALT may coordinately contribute to immune surveillance on the ocular surface.

In conclusion, we identified M cells in the lacrimal sac of mice by detecting molecular markers common to intestinal M cells. These molecules include several receptors for bacterial pathogens as well as molecules regulating receptor activation, suggesting that TALT M cells contribute to the immune response against infection of the ocular mucous membranes. Together with the newly developed technique of separating TALT, our research will help understand ocular mucosal immunity.

## Data Availability Statement

The raw data supporting the conclusions of this article will be made available by the authors, without undue reservation.

## Ethics Statement

The animal study was reviewed and approved by Animal Experimentation at the Animal Use Committee at the Keio University.

## Author Contributions

YO: methodology, investigation, and writing–original draft. SK: conceptualization and writing–review and editing. YN NI, ST, RM, and ME: methodology and investigation. KH: writing–review, and editing. SK and KH: supervision and funding acquisition.

## Funding

This work was supported by JSPS KAKENHI grant number 19K07239 (SK), 20H05876, 20H00509 (KH), JST PRESTO grant number 19199152 (SK), JST CREST grant number JPMJCR19H1 (KH), JST Moonshot R&D grant number JPMJMS2025 (KH), and AMED CREST grant number 21gm1010004h0106 and 21gm1310009h0002 (KH).

## Conflict of Interest

The authors declare that the research was conducted in the absence of any commercial or financial relationships that could be construed as a potential conflict of interest.

## Publisher’s Note

All claims expressed in this article are solely those of the authors and do not necessarily represent those of their affiliated organizations, or those of the publisher, the editors and the reviewers. Any product that may be evaluated in this article, or claim that may be made by its manufacturer, is not guaranteed or endorsed by the publisher.
