# Supplementary material for: Characterization of M Cells in Tear Duct-Associated Lymphoid Tissue of Mice: A Potential Role in Immunosurveillance on the Ocular Surface
Source: Front Immunol. 2021 Nov 22;12:779709. doi: 10.3389/fimmu.2021.779709 (PMC8645900; doi:10.3389/fimmu.2021.779709)
Supplement: Supplementary file 1 [file DataSheet_1.pdf]

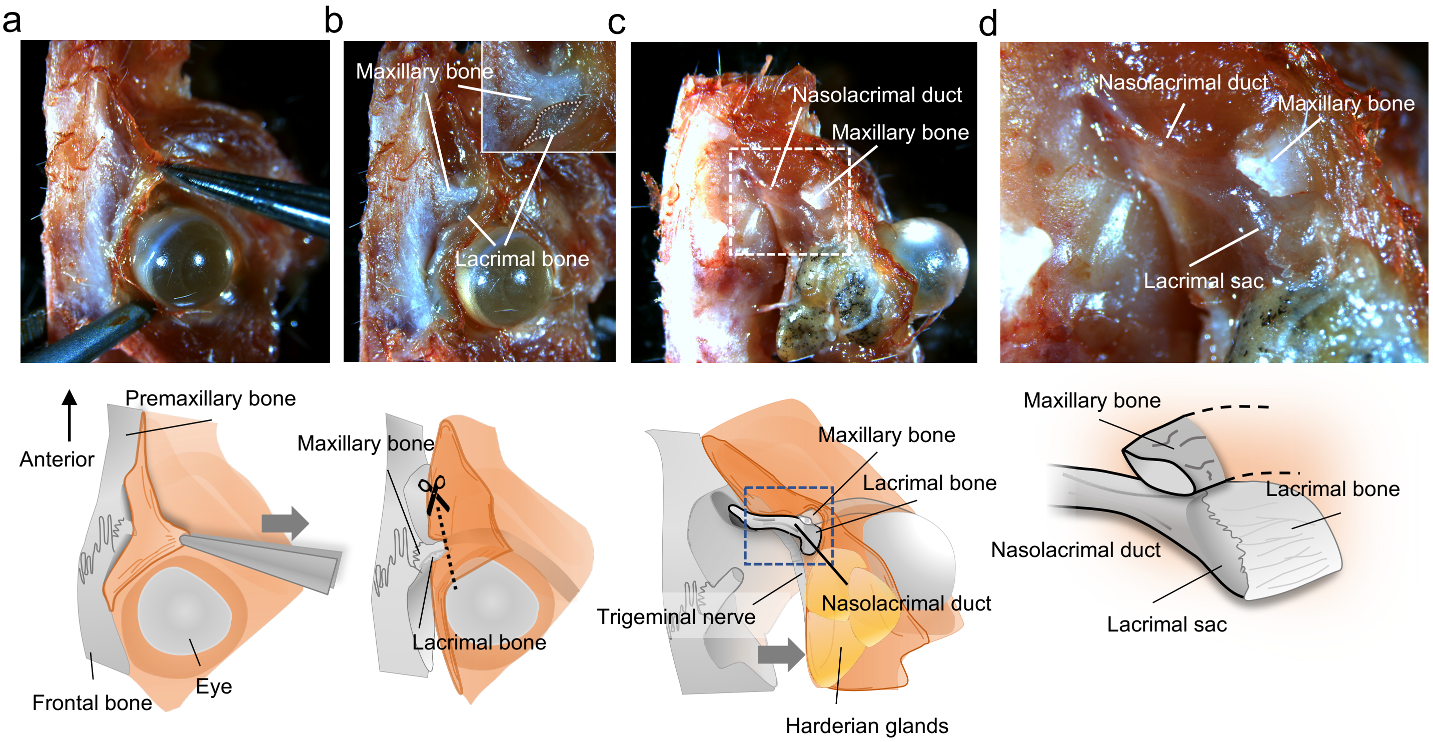

Supplementary Figure 1

The preparation of nasolacrimal duct from mouse head. The head was amputated in half along the midline and the incisor and hard palate were removed by scissor. This is an illustration for isolating of the nasolacrimal duct of the right eye. Frontal, premaxillary, maxillary, and lacrimal bones was exposed by a precision tweezer No.5 (a, b). Subsequently, the maxillary bone was carefully amputated with scissors so as not to damage the lacrimal bone and the associated lacrimal sac (b). The nasolacrimal duct was then exposed by carefully peeling off the face from bones (c, d).

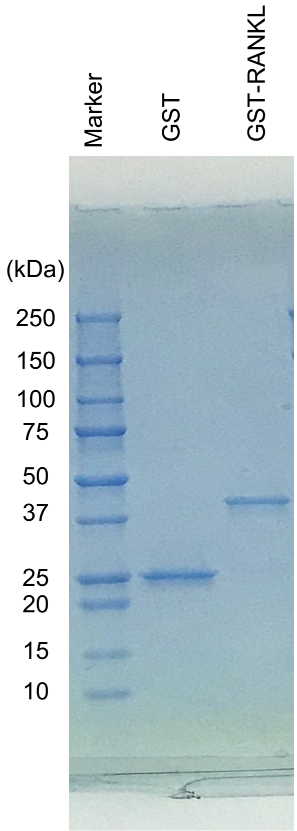

Supplementary Figure 2
Purified GST (25 kDa) and GST-RANKL (40 kDa) proteins (1.25 µg/lane) were separated by SDS-PAGE and stained with CBB.

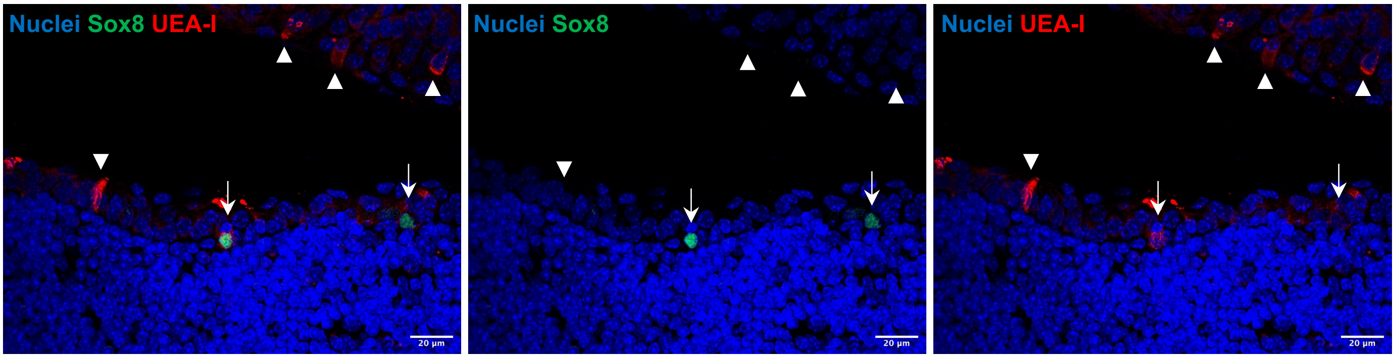

Supplementary Figure 3
Tissue sections prepared from the nasolacrimal duct including TALT were stained with anti-Sox8 antibodies (green) and UEA-I (red). Nuclei were stained with Hoechst 33342 (blue). UEA-I+ Sox8+ cells were found in FAE (arrow), whereas UEA-I+ Sox8- cells were found both in FAE and outside of FAE (arrowheads). Bars: 20 µm.

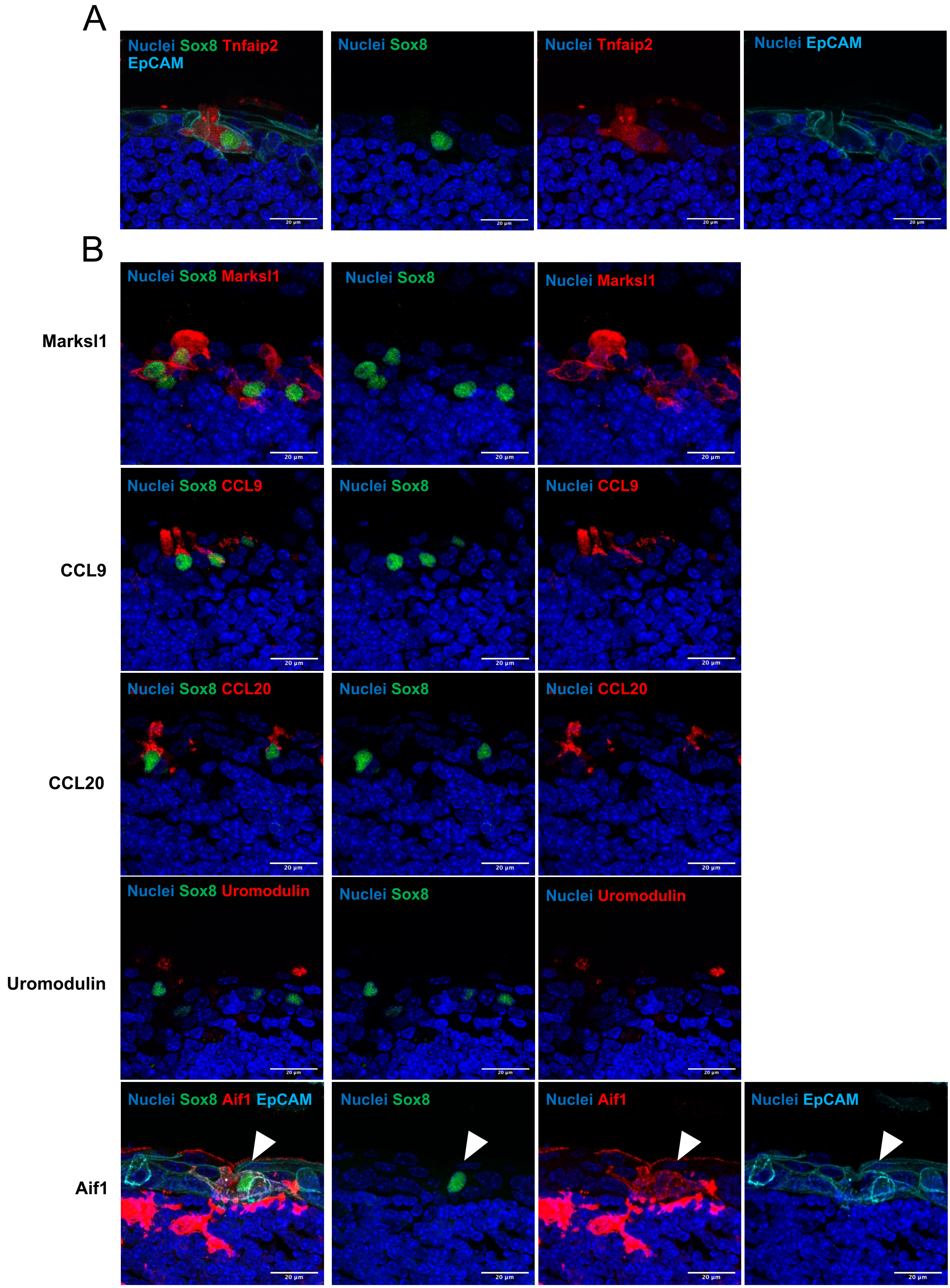

Supplementary Figure 4

(A) Sox8 (green) Tnfaip2 (red) double-positive cells in the TALT FAE simultaneously express EpCAM (cyan) that is an epithelial cell marker. Nuclei were counterstained with Hoechst 33342 (blue). Bars: 20μm. n = 3 animals. (B) Confocal microscopic images of TALT FAE prepared through cryosections and stained with indicated antibodies in each column. Sox8 (Green) and respective M cell markers (Red): Marksl1, CCL9, CCL20, Uromodulin, and Aif1. Arrowheads show a $Sox8^{+}$ $Aif1^{+}$ $EpCAM^{+}$ cell. Nuclei were stained with Hoechst 33342 (blue). Bars: 20μm. Representative images of those obtained from at least 3 animals are shown.

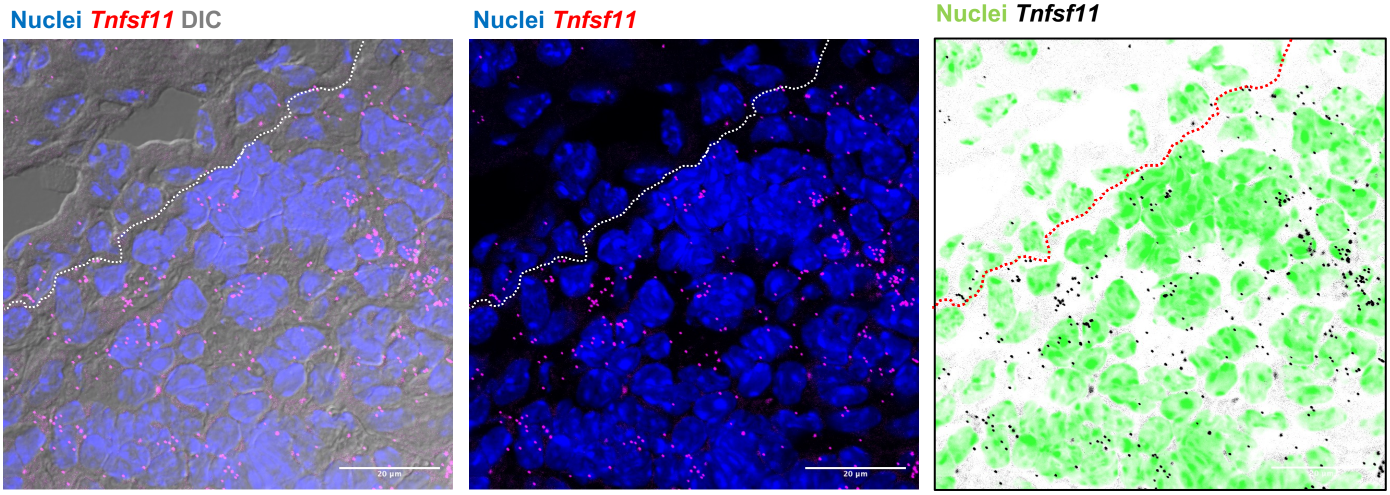

Supplementary Figure 5

Fluorescence in situ hybridization (FISH) images of TALT FAE of BALB/c mice using oligonucleotide probes for *Tnfsf11* encoding RANKL. One image is represented by different pseudo-colors; the locations of *Tnfrsf11b* are represented by magenta dots on the left and the center panels and black dots on the right panel. Nuclei are blue in the left and center panels and green in the right panel. Basal membranes are shown by white line in the left and the center panel, and red line in the right panels. DIC (gray); differential interference contrast. Bars: 20 µm. Representative images of those obtained from at least 3 animals are shown.

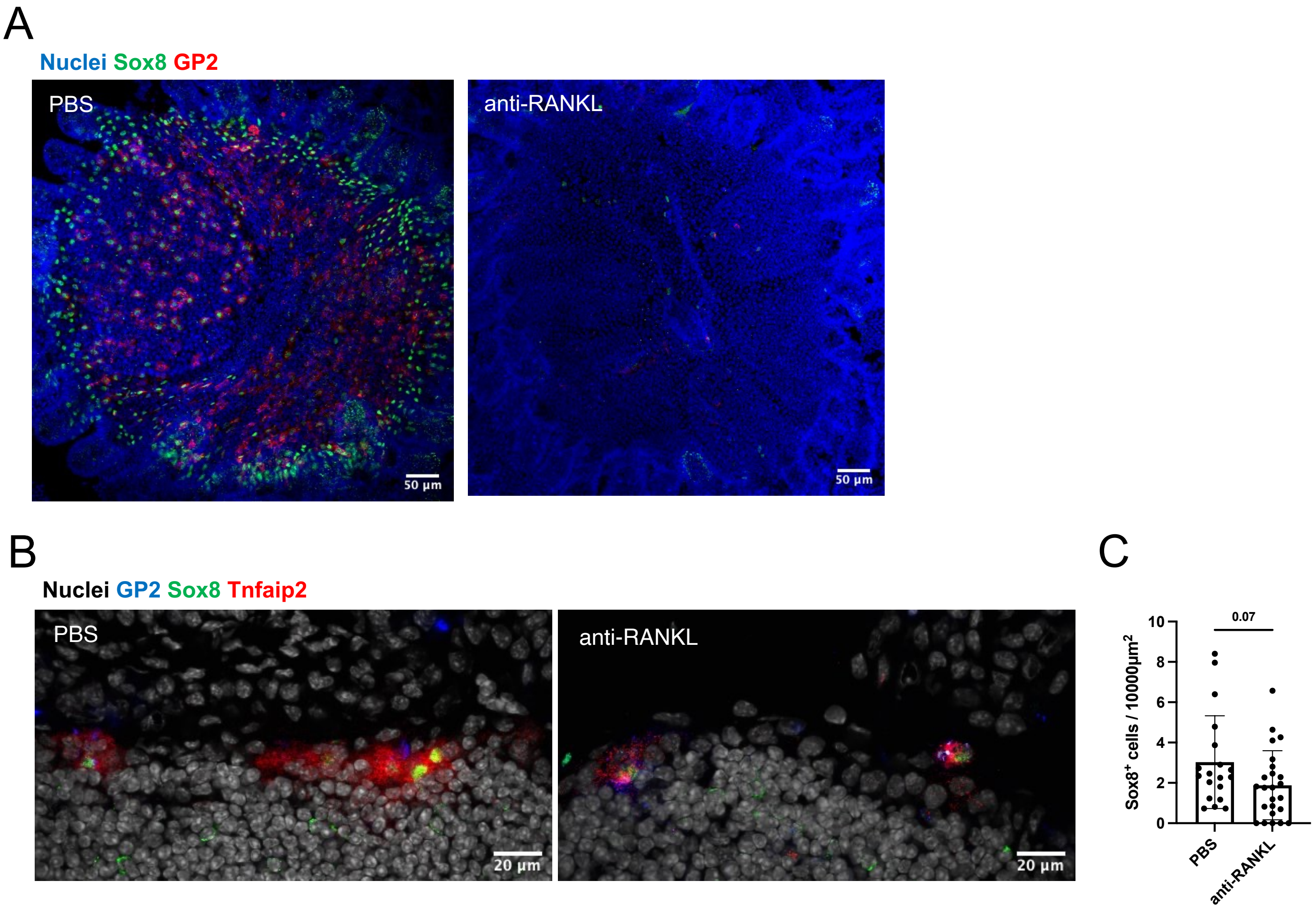

Supplementary Figure 6

Mice were intraperitoneally injected with anti-RANKL antibodies two times every four days. Four days after second injection, mice were euthanized and were subjected to immunofluorescence analysis. (A) Whole-mount immunostaining of the FAE of Peyer's patches for Sox8 (green) and GP2 (red). Nuclei were stained with Hoechst 33342 (blue). Bars: 20 μm. (B) The expression of GP2 (blue), Sox8 (green) and Tnfaip2 (red) in TALT FAE. Nuclei were stained with Hoechst 33342 (gray). Bars: 20 μm. (C) Quantification of $Sox8^{+}$ cells in the TALT FAE. Data were presented as the mean ± standard deviation. Student's t-test, n = 3 animals, respectively. Each symbol represents the number of $Sox8^{+}$ cells per 10000 μm^2 of cryosections.

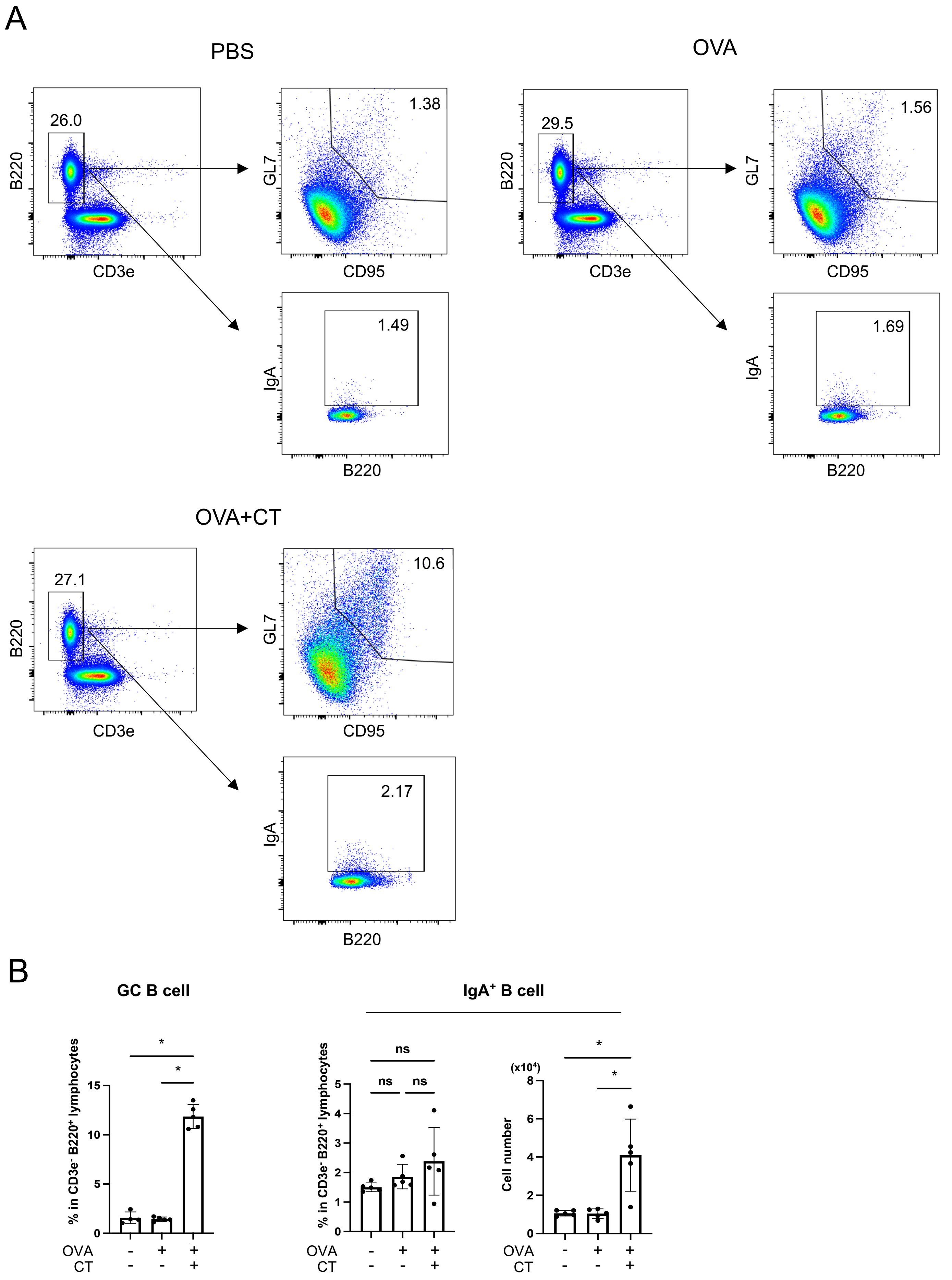
A
PBS
OVA
OVA+CT
B220
CD3e
GL7
CD95
IgA
26.0
1.38
1.49
29.5
1.56
1.69
27.1
10.6
2.17
B
GC B cell
IgA⁺ B cell
% in CD3e⁻ B220⁺ lymphocytes
Cell number
(x10⁴)
ns
*
OVA
CT
- - +
- - +
- + +

C

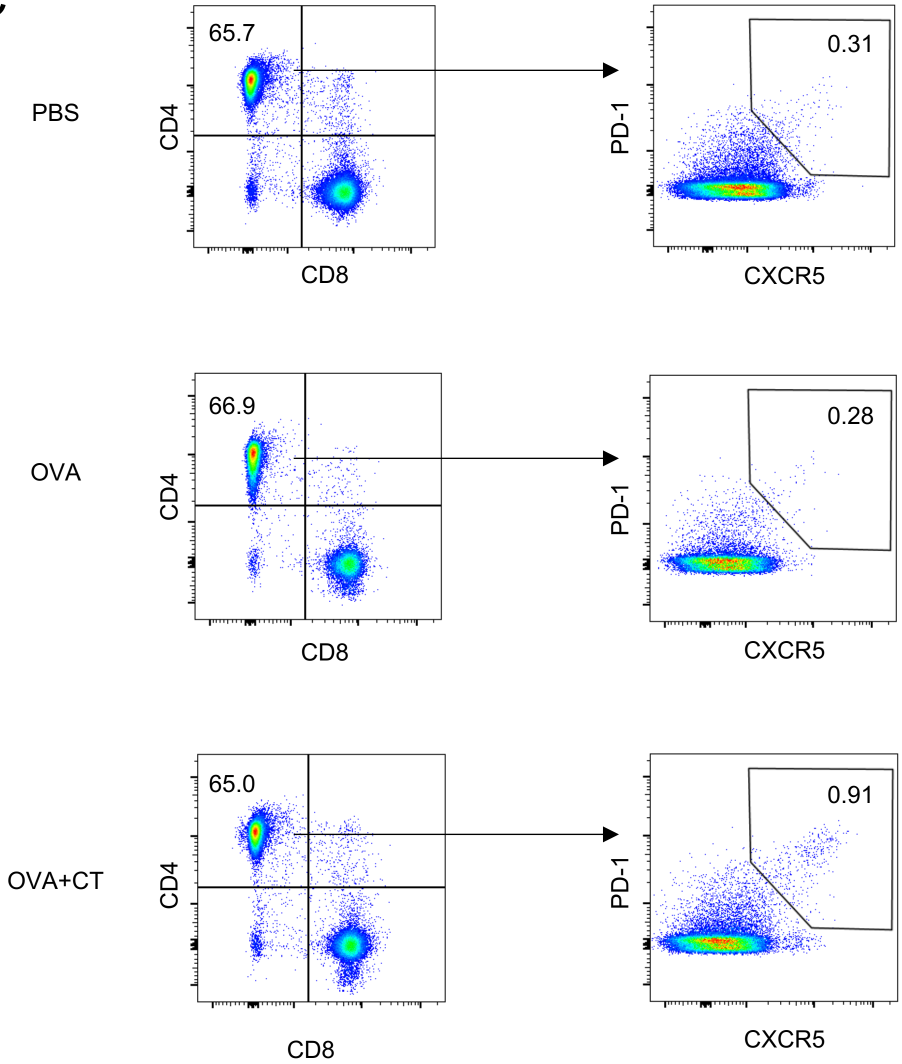

D

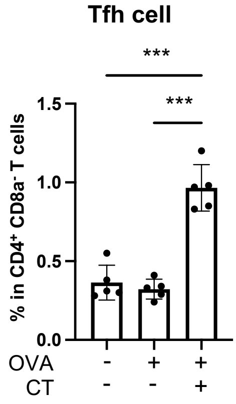

Supplementary Figure 7

FACS sorting strategies for subsets of GC B cells &IgA$^+$ B cells (A, B) and Tfh cells (C, D). Numbers adjacent to outlined areas in right panels show proportion of GC B cells or IgA$^+$ B cells in CD3e$^-$ B220$^+$ lymphocytes or Tfh cells in CD4$^+$ CD8$^-$ T cells in the cervical and submandibular lymph nodes (cLN and smLN). Data are presented the mean ± standard deviation. *** p < 0.001, * p <0.05, ns; not significant (GC B cell and IgA$^+$ B cell: Kruskal-Wallis test, Tfh cell: one-way ANOVA by Tuker's test, PBS: n = 4 or 5, OVA: n = 5, OVA+CT: n = 5 animals)

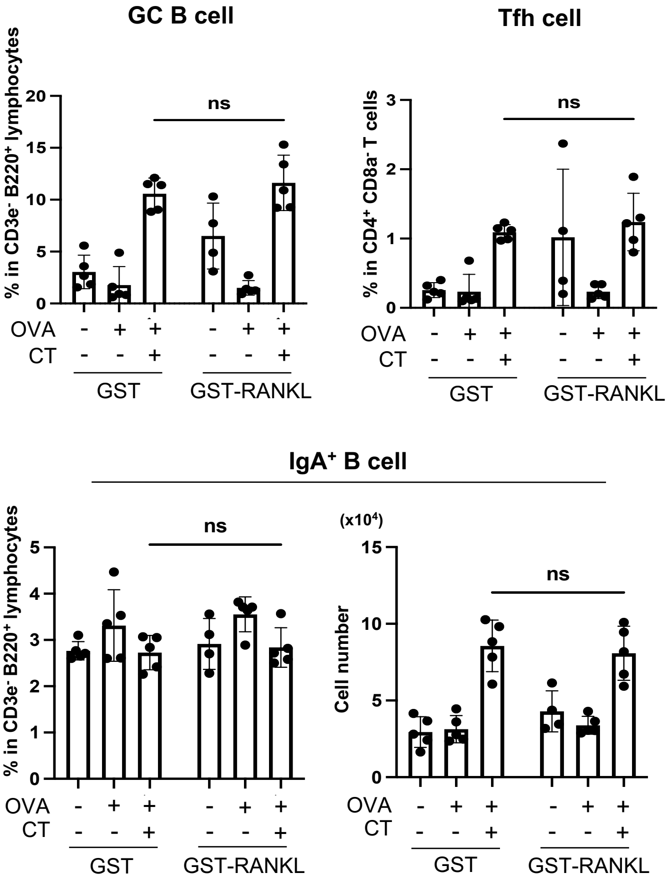

Supplementary Figure 8

The frequencies of GC B cells in CD3e$^-$ B220$^+$ lymphocytes and Tfh cells in CD8a$^-$ CD4$^+$ T cells in cLN and smLN from BALB/c mice injected with GST or GST-RANKL daily for 3 days (first and third week: 5 mg/kg, second week: 2.5 mg/kg) before instillation of OVA plus CT by eye drops. Data are representative from two independent experiments and are presented as the mean ± standard deviation. ns; not significant (two-way ANOVA, PBS/GST: n = 5, OVA/GST: n = 5, OVA+CT/GST: n = 5, PBS/GST-RANKL: n = 4, OVA/GST-RANKL: n = 5, OVA+CT/GST-RANKL: n = 5 animals).

Supplementary Table 1 : List of the antibodies and lectin for immunostaining analysis

**Primary antibodies**	**Host**	**Dilution**	**Source**
anti-B220	rat	1:200	BioLegend; clone RA3-6B2
anti-Sox8	guinea pig	1:2000	Generated in-house (17)
anti-GP2	rat	1:200	MBL; clone 2F11-C3
anti-Tnfaip2	rabbit	1:200	Generated in-house (24)
anti-CCL9	goat	1:100	R&D systems; AF463
anti-CCL20	goat	1:200	R&D systems; AF760
anti-OPG	goat	1:400	R&D systems; AF549
anti-Aif1	goat	1:200	abcam; ab5076
anti-Marksl1	rabbit	1:200	Proteintech; 10002-2-AP
anti-Uromodulin	sheep	1:200	R&D systems; AF5175
anti-Spi-B	sheep	1:200	R&D systems; AF7204
anti-ZO-1	rabbit	1:200	Thermo fisher Scientific; 61-7300
anti-RANKL	guinea pig	1:1000	Generated in-house
anti-RANK	goat	1:400	R&D systems; AF692
anti-EpCAM	rat	1:200	eBiosciences; clone G8.8
**Secondly antibodies**	**Host**	**Dilution**	**Source**
HRP anti-rat IgG	goat	1:1000	Thermo fisher Scientific; 31470
Alexa Fluor 488 anti-rat IgG	donkey	1:800	Jackson ImmunoResearch; 712-545-153
Alexa Fluor 488 anti-guinea pig IgG	donkey	1:800	Jackson ImmunoResearch; 706-545-148
Cy3 anti-sheep IgG	donkey	1:800	Jackson ImmunoResearch; 713-165-147
Cy3 anti-guinea pig IgG	donkey	1:800	Jackson ImmunoResearch; 706-165-148
Cy3 anti-goat IgG	donkey	1:800	Jackson ImmunoResearch; 705-165-147
Cy5 anti-rat IgG	donkey	1:800	Jackson ImmunoResearch; 712-175-153
Alexa Fluor 488 anti-rabbit IgG	donkey	1:800	Thermo fisher Scientific; A32790
Alexa Fluor 555 anti-rabbit IgG	donkey	1:800	Thermo fisher Scientific; A32794
Alexa Fluor 647 anti-rabbit IgG	donkey	1:800	Thermo fisher Scientific; A32795
**A Lectin**	**Host**	**Dilution**	**Source**
Rhodamine-conjugated UEA I	-	1:200	VECTOR laboratories; RL-1062-2
Alexa Fluor 488 PNA	-	1:400	Thermo fisher Scientific; L21409

Supplementary Table 2: List of the oligonucleotide primers

Target gene	sequence
Gapdh	Forward 5' -AGGTCGGTGTGAACGGATTTG- 3'<br>Reverse 5' -TGTAGACCATGTAGTTGAGGTC- 3'
Sox8	Forward 5' -CGAGGGGATACTGCTGAGG-3'<br>Reverse 5' -AGCTCTGCGTTATGGAGATGC- 3'
Spib	Forward 5' -CCGAGGGGAGGGGATCTGAG- 3'<br>Reverse 5' -GGAGGAGAACTGGAAGACGCCG- 3'
Tnfaip2	Forward 5' -GTGCAGAACCTCTACCCCAATG- 3'<br>Reverse 5' -TGGAGAATGTCGATGGCCA- 3'
Tnfrsf11b	Forward 5' -ACCCAGAAACTGGTCATCAGC- 3'<br>Reverse 5' -CTGCAATACACACACTCATCACT- 3'
Gp2	Forward 5' -GATACTGCACAGACCCCTCCA- 3'<br>Reverse 5' -GCAGTTCCGGTCATTGAGGTA - 3'
Tnfrsf11a	Forward 5' -CCAGGAGAGGCATTATGAGCA- 3'<br>Reverse 5' -ACTGTCGGAGGTAGGAGTGC- 3'
Ccl9	Forward 5' -CAACAGAGACAAAAGAAGTCCAGAG- 3'<br>Reverse 5' -CTTGCTGATAAAGATGATGCCC- 3'
Ccl20	Forward 5' -TGCTCTTCCTTGCTTTGGCATGGGTA- 3'<br>Reverse 5' -TCTGTGCAGTGATGTGCAGGTGAAGC- 3'
Aif1	Forward 5' -TGAGGAGATTTCAACAGAAGCTGA- 3'<br>Reverse 5' -CCTCAGACGCTGGTTGTCTT- 3'

Supplementary Table 3 : List of the antibodies for Flow cytometry

Antibodies and Streptavidin	Dilution	Source
Biotin anti-IgA	1:100	BD Biosciences; 556978
Streptavidin FITC Conjugate	1:400	eBioscience; 11-4317-87
Alexa Fluor 647 anti-GL7	1:100	BioLegend; 144606
APC-R700 anti-CD95	1:100	BD Biosciences; 565130
Brilliant Violet 510 anti-CD45	1:400	BioLegend; 103138
APC-H7 anti-B220	1:400	BD Biosciences; 565371
PE anti-CD3e	1:400	eBiosciences; 12-0031-83
APC anti-CXCR5	1:50	BioLegend; 145506
Brilliant Violet 421 anti-PD-1	1:100	BioLegend; 135221
Red Fluor 710 anti-CD4	1:400	TONBO Biosciences; 80-0042-U100
FITC anti-CD8a	1:400	TONBO Biosciences; 35-0081-U500
